# Tumor-infiltrating macrophages and dendritic cells in human colorectal cancer: relation to local regulatory T cells, systemic T-cell response against tumor-associated antigens and survival

**DOI:** 10.1186/1479-5876-5-62

**Published:** 2007-11-29

**Authors:** Dirk Nagorsen, Sabine Voigt, Erika Berg, Harald Stein, Eckhard Thiel, Christoph Loddenkemper

**Affiliations:** 1 Department of Hematology and Oncology, Charité – Universitätsmedizin Berlin, Campus Benjamin Franklin, Berlin, Germany; 2 Department of Pathology, Charité – Universitätsmedizin Berlin, Campus Benjamin Franklin, Berlin, Germany; 3 Research Center ImmunoSciences (RCIS), Charité – Universitätsmedizin Berlin, Berlin, Germany

## Abstract

**Introduction:**

Although systemic T-cell responses against tumor antigens and tumor infiltration by T cells have been investigated in colorectal cancer (CRC), the initiation of spontaneous immune responses *in situ *is not well understood. Macrophages and dendritic cells (DC) play an important role as a link between innate and adaptive immune response. The aim of the present study was to analyze macrophage and DC infiltration in CRC and to investigate whether there is a correlation to systemic T-cell response, regulatory T cell (Treg) infiltration, and survival.

**Methods:**

Immunohistological staining was performed with nine markers for macrophages and DC (CD68, CD163, S100, CD11c, CD208, CD209, CD123, CD1a, Langerin) in 40 colorectal cancer samples from patients, in whom the state of systemic T-cell responses against tumor-associated antigens (TAA) and Treg infiltration had previously been determined.

**Results:**

All specimens contained cells positive for CD68, CD163, S100 and CD1a in epithelial tumor tissue and tumor stroma. Only a very few (less than median 3/HPF) CD123+, CD1a+, CD11c+, CD 208+, CD209+, or Langerin+ cells were detected in the specimens. Overall, we found a trend towards increased infiltration by S100-positive DC and a significantly increased number of stromal S100-positive DC in patients without T-cell response. There was an increase of stromal S100 DC and CD163 macrophages in limited disease (S100: 11.1/HPF vs. 7.3/HPF, p = 0.046; CD163: 11.0/HPF vs. 8.1/HPF, p = 0.06). We found a significant, positive correlation between S100-positive DC and FOXP3-positive Tregs. Survival in patients with high DC infiltration was significantly better than that in those with low DC infiltration (p < 0.05). Furthermore, we found a trend towards better survival for increased infiltration with CD163-positive macrophages (p = 0.07).

**Conclusion:**

The present *in situ *study adds new data to the discussion on the interaction between the innate and adoptive immune system. Our data strongly support the hypothesis that tumor-infiltrating DC are a key factor at the interface between innate and adaptive immune response in malignant disease. Tumor infiltrating S100-positive DC show an inverse relationship with the systemic antigen-specific T-cell response, a positive correlation with regulatory T cells, and a positive association with survival in CRC. These data put tumor-infiltrating DC at the center of the relevant immune response in CRC.

## Introduction

Colorectal cancer (CRC) is a common malignant disease, which has been intensely studied for tumor-immune interactions to develop successful immunotherapies. In particular, systemic T-cell responses against tumor antigens and tumor-infiltrating T cells have been analyzed in detail in CRC. Several investigators have linked a high T cell infiltration to a better survival in CRC [1-6]. Patients with CRC – as well as those with other malignant diseases – are able to mount an antigen-specific T cell response without prior immunotherapy [7,8]. In a first analysis with a limited number of colorectal cancer patients, no survival benefit was found for patients with peripheral TAA-directed T-cell responses [9]. Various components including the immune system, tumor stroma and tumor cells themselves influence the induction and modulation of tumor-directed immune responses [10]. Limited anti-tumor activity of spontaneous antigen-specific T cells at a clinical level in CRC patients may be due to multiple factors.

Approximately 20 years ago, mononuclear phagocytes were described for the first time as being increased in CRC compared to healthy tissue [11]. This observation has been confirmed several times, for example, just recently by Sickert and co-workers [12]. Furthermore, it has been known for about the same amount of time that high numbers of tumor-associated macrophages (TAM) and tumor-associated DC (TADC) are related to a better survival in CRC [13-15]. This also agrees with data in other malignant diseases [16,17]. For tumor-infiltrating macrophages, it is controversially discussed in the literature whether a trend towards high macrophage infiltration in CRC is a positive prognostic factor [3,5,18-21].

Dendritic cells, i.e. professional antigen-presenting cells, play a critical role in innate and adaptive immune responses [22]. In the development of spontaneous T-cell responses, the interaction between DC and other compartments of the immune system (Tregs, T cells) is of central importance. In a previous work, we showed that the average Treg infiltration rate in the tumor was slightly higher in patients without systemic TAA-specific T-cell response and that Treg infiltration in the tumor stroma was significantly higher in limited disease than in metastatic CRC [23]. Understanding the role of resident DC in relation to local Tregs and systemic immune response might offer great potential in the context of active-specific vaccination against cancer and the mediation of systemic immunotherapies at the tumor site [24-26].

The aim of the present study was to analyze colorectal cancer infiltration by macrophages and dendritic cells of various phenotypes *in situ *and to test whether there is a correlation to a systemic TAA-specific T-cell response, local infiltration by regulatory T cells, disease stage and survival of CRC patients.

## Methods

### Patient selection, immunohistochemistry and T cell assays

After institutional review board approval and informed consent, peripheral blood mononuclear cells from CRC patients were collected and frozen for T cell analysis. All analyses were performed in compliance with the Helsinki Declaration. HLA-A2-positive patients were tested for the presence of T-cell responses against the HLA-A*0201-presented T cell epitopes Ep-CAM p263-271, her-2/*neu *p654-662, and CEA p571-579 by ELISPOT assay. HLA analysis and ELISPOT were performed as previously described [7,8]. Positive T-cell responses were also defined as previously described [7-9]. Immunohistochemistry for FOXP3, CD3, and CD8 had been performed on the same patient samples earlier [23]. Sufficient tumor sections for immunohistochemistry and sufficient clinical cancer were required for inclusion.

### Immunohistochemistry and microscopic analysis

For immunostaining, 4 μm thick sections were cut, de-paraffinized, and subjected to heat-induced epitope retrieval before incubation with antibodies. For this purpose, sections were immersed in sodium citrate buffer at pH 6.0 and heated in a high-pressure cooker. After cooking, the slides were rinsed in running water, washed with Tris-buffered saline, pH 7.4 and incubated with the primary antibodies. All primary antibodies employed are listed in Table 1. For double labelling S100/CD163, slides were incubated with the polyclonal rabbit antibody against S100 protein, blocked using a commercial peroxidase-blocking reagent and developed using the streptavidin peroxidase kit (Dako, Glostrup, Denmark). Sections were then incubated with the mouse monoclonal antibody against CD163, and the streptavidin AP kit (Dako) was used for detection. Alkaline phosphatase was developed using Fast Red as the chromogen, while peroxidase was visualized with diaminobenzidine chromogen as the substrate. Tonsillar tissue with follicular hyperplasia served as positive controls, and negative controls were performed by omitting the primary antibodies. Ten randomly chosen high power fields (1 HPF = 0,237 mm^2^) were analyzed for macrophage or dendritic cell infiltration in the tumor tissue and the tumor stroma and averaged in each case.

**Table 1:** Panel of antibodies used in this study

**Antibody**	**Clone**	**Antigen retrieval**	**Dilution**	**Main cell reactivity**	**Source**
CD68	PGM1	Citrate	1:50	Macrophages	Dako, Glostrup, Denmark
CD163	10D6	Citrate	1:100	Macrophages	Novocastra, Newcastle upon Tyne, UK
S100	rabbit polyclonal	Citrate	1:500	DC	Dako
CD11c	5D1	Citrate	1:50	interdigitating DC, stromal DC	Novocastra
DC-LAMP/CD208	104.G4	Protease	1:50	mature DC	Immunotech, Marseille Cedex, France
DC-SIGN/CD209	DCN46	Citrate	1:100	stromal DC	BD Pharmingen, San Diego, CA, USA
CD123	9F5	Citrate	1:10	plasmacytoid DC	BD Pharmingen
CD1a	010	Citrate	1:50	Langerhans cells, interdigitating DC	Dako
Langerin/CD207	12D6	Citrate	1:100	Langerhans cells	Novocastra

### Statistical and survival analysis

#### TAM/TADC infiltration and systemic T-cell response

Patients were sorted according to their positive or negative peripheral T-cell response. The infiltration with TAM/TADC in these two groups was compared using the Student's t-test, separately for epithelial and stromal infiltration.

#### TAM/TADC infiltration and survival

All patients were sorted according to their TAM or TADC infiltration. The median was used to separate patients with high and low infiltration. A Kaplan-Meier survival analysis was performed comparing patients with high and low infiltration. A log-rank test was performed to test statistical significance.

#### Stage correction

We then compiled patient groups according to their disease stage to reduce the influence of external factors. Stage correction was performed by grouping patients by their UICC stage. We combined UICC I and II because only one patient was UICC I. These stage-grouped patients were sorted by TAM/TADC infiltration. The median was again used to separate patients with infiltration lower or higher than the median. All patients with low infiltration in stage group I+II, III, and IV were combined into a new "stage-corrected low infiltration group". A "stage-corrected high infiltration group" was similarly defined. These two groups were then compared using Kaplan-Meier survival analysis. A log-rank test was performed to test statistical significance.

#### Association of TAM/TADC infiltration and stage, age, and gender

To analyze differences concerning TAM/TADC infiltration and stage, patients were grouped according to limited (UICC I and II) and metastatic (UICC III and IV) disease. These two groups were compared for tumor and stromal infiltration by TAM and TADC using the Student's t-test. We also tested the association between TAM/TADC infiltration and age (Pearson's correlation) and gender (t-test).

#### Correlation of TAM/TADC with tumor-infiltrating T cells

Pearson's correlation was calculated for TAM and TADC infiltration with tumor-infiltrating Tregs, CD3+ T cell, and CD8+ T cell infiltration and separately for stromal and epithelial tumor infiltration. Since correlations between the various tumor-infiltrating immune cells have to be considered descriptive due to multiple testing, we additionally performed a Bonferroni correction.

A level of p < 0.05 was considered significant and p < 0.1 a trend. SPSS software was used.

## Results

### Patient characteristics

Forty CRC patients met all inclusion criteria. These are the same 40 patients previously published for their Treg infiltration [23]. One patient had UICC I, 20 patients UICC II, six patients UICC III, and 13 patients UICC IV. Eleven patients tested positively for a systemic TAA-specific T-cell response to HLA-A2-binding peptides of CEA, Ep-CAM, and/or her-2/neu. Twenty-one patients were female and nineteen male. Mean age at first diagnosis was 62.1 years (patients with T-cell response: 61.7 years, patients without T-cell response: 62.2 years).

### General description of macrophage and DC infiltration

**Figure 1:**
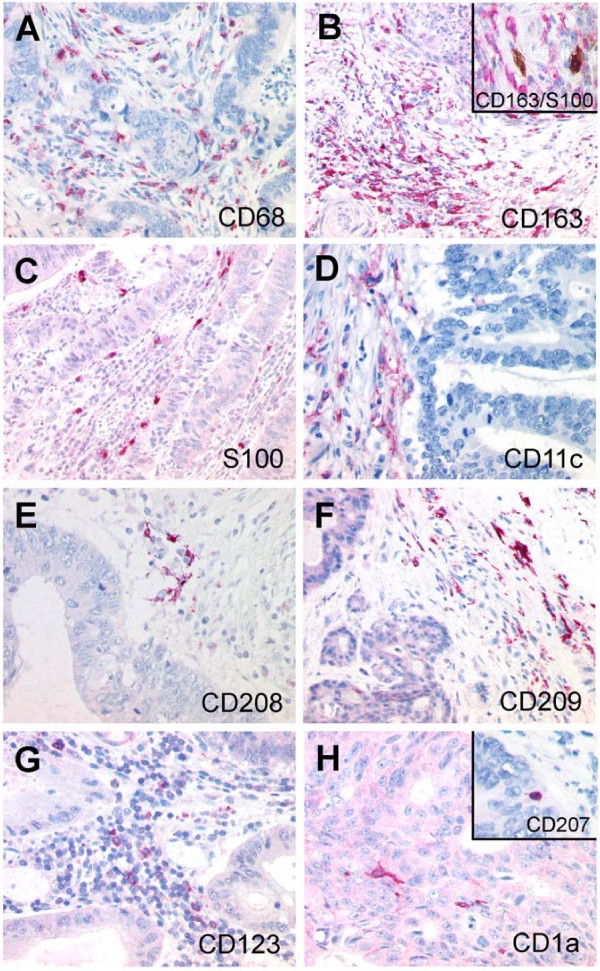
Immunohistochemical staining of macrophage and dendritic cell infiltration in colorectal cancer. (A) CD68 and (B) CD163 expression of the macrophages (inset: double labeling CD163 (red)/S100 (brown) distinguishes CD163+ macrophages from S100+ DC). Dendritic cell populations with expression of (C) S100, (D) CD11c, (E) CD208, (F) CD209, (G) CD123 and (H) CD1a (inset: Langerin/CD207). (magnification ×200, inset ×400).

We found an infiltration of tumor stroma and epithelial tumor tissue by CD68- and CD163-positive macrophages and S100-expressing dendritic cells in all 40 specimens as summarized in Table 2. The frequency of CD68-, S100-, CD163-, and CD209-positive cells was significantly higher in stromal than in epithelial tumor tissue. Langerin-, CD208-, and CD123-positive cells were very rare. Examples are given in Figure 1.

**Table 2:** Infiltration of cells expressing DC or macrophage markers

	CD68	CD163	S100	CD11c	CD208	CD209	CD123	CD1a	Langerin
Mean cell number per HPF ± SD	15.9 ± 8.8	12.4 ± 6.3	11.5 ± 7.3	1.8 ± 2.4	1.0 ± 1.5	2.8 ± 3.2	0.6 ± 1.0	2.2 ± 2.8	0.1 ± 0.3
Range	4.0 – 52.0	3.9 – 30.0	1.7 – 46.0	0- 11.7	0 – 7.0	0.1 – 16.1	0 – 5.5	0.2 – 12.4	0 – 1.1
At least 0.2 cells per HPF in % of samples	100%	100%	100%	95%	77.5%	97.5%	70%	100%	32.5%
Stromal infiltration cells/HPF	12.8*	9.7*	9.3*	1.3	0.6	2.2*	0.3	1.1	0.1
Epithelial infiltration cells/HPF	3.1	2.8	2.2	0.5	0.3	0.6	0.4	1.1	0.1

(Differences due to rounding error, * significantly increased compared to epithelial infiltration)

### Double staining of CD163 and S100

Double staining revealed that approximately 20% of S100-positive cells were also positive for CD163, indicating that, with our staining protocol, only a relatively small percentage of macrophages were also labeled with the anti-S100 antibody and only S100+/CD163- cells were regarded as DC.

### Macrophage/DC infiltration and systemic T-cell response

There is a trend towards increased infiltration by S100-positive dendritic cells in patients without systemic T-cell response compared to those with systemic T-cell response (12.8/HPF vs. 8.1 HPF, p = 0.07). This trend is due to significantly increased stromal infiltration in patients without T-cell response (10.6/HPF vs. 5.9/HPF, p = 0.03). There was also a trend towards increased CD11c infiltration in patients with systemic T-cell response (3.0 vs. 1.4, p = 0.05). Cell infiltration determined by the other markers was not different between patients with and without systemic T-cell response.

### Macrophage/DC infiltration and stage of colorectal cancer

There is no single staining which is significantly increased or decreased with regard to the stage in our study population (total cell numbers). In a subset analysis, there was an increase of stromal S100+ DC and CD163-positive macrophages in limited disease (S100: 11.1/HPF vs. 7.3/HPF, p = 0.046; CD163: 11.0/HPF vs. 8.1/HPF, p = 0.06). Additionally, there was a trend toward increased CD11c infiltration in advanced disease (0.13/HPF vs. 0.98/HPF, p = 0.08).

### Macrophage/DC infiltration and T cell/Treg infiltration

The correlation coefficients between various cell subsets are listed in Table 3. Data on T cell infiltration have been published previously [23]. After Bonferroni correction, we found that the following pairs significantly correlated with each other: CD123 – CD68, S100 – FOXP3, CD1a – CD68, CD208 – FOXP3, and not surprisingly CD3 – CD8 and FOXP3 – CD3. Due to the fact that CD123, CD1a, and CD208 are found only at very low infiltration levels, we regard the positive correlation between S100-positive DC and FOXP3-positive Treg (Pearson's correlation coefficient 0.59, p < 0.001) the most important.

**Table 3:** Pearson's correlation coefficients between TAM/TADC and other tumor infiltrating immune cells

	CD68	S100	CD11c	CD163	CD208	CD209	CD1a	FOXP3	CD3	CD8
CD123	0.541***	n.s.	n.s.	n.s.	n.s.	n.s.	0.331*	n.s.	n.s.	0.317*
CD68	1	0.361*	n.s.	n.s.	n.s.	n.s.	0.548***	n.s.	n.s.	n.s.
S100		1	n.s.	n.s.	0.449**	n.s.	n.s.	0.592***	n.s.	n.s.
CD11c			1	n.s.	0.475**	n.s.	n.s.	n.s.	n.s.	n.s.
CD163				1	n.s.	0.343*	0.379*	n.s.	0.457**	0.399*
CD208					1	n.s.	n.s.	0.612***	0.372*	n.s.
CD209						1	0.342*	n.s.	n.s.	n.s.
CD1a							1	n.s.	n.s.	n.s.
FOXP3								1	0.657***	0.449**
CD3									1	0.930***

n.s. not significant, *p < 0.05, ** p < 0.01, ***p < 0.001

### Macrophage and DC infiltration and survival

**Figure 2:**
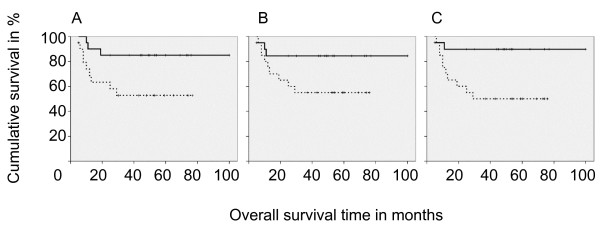
Kaplan Meier survival analyses of patients with high (solid) and low (dashed) cell infiltration in the tumor. A) Patients with high S100 infiltration have a significantly better survival (p = 0.03). B) Patients with high total CD163 tend to have a better survival (p = 0.07). C) Patients with high stromal CD163 infiltration have a significantly better survival (p = 0.01).

We found significantly better survival in patients with high infiltration by S100-positive DC (p = 0.03, Figure 2A). To exclude a dominant role of S100+ macrophages, not only did we calculate survival in relation to S100+ but also CD163-negative cell infiltration (calculated from double staining). This calculation confirmed a better survival for patients with high DC infiltration (p = 0.047). This better survival was also found for S100+ DC infiltration of tumor stroma but not epithelial tissue. After stage correction there was still a strong trend towards better survival of patients with higher S100-positive DC infiltration (p = 0.06). Moreover, we found a trend towards better survival for increased infiltration with CD163-positive macrophages (p = 0.07, Figure 2B). This trend was also confirmed after stage correction (p = 0.07). Patients with high stromal CD163+ macrophage infiltration survived significantly longer (p = 0.01, Figure 2C).

### Macrophage/DC infiltration and age and gender

We found a difference between male and female patients for CD163+ cells. Female patients had 14.5/HPF and male patients 10.2/HPF (p = 0.03) macrophages. None of the other markers showed any differences between males and females. A weak trend towards a negative correlation was seen between age and stromal S100 DC infiltration (Pearson's = -0.332, p = 0.05). No relation to age was found for the other infiltrating cell populations. It should be noted that age had no impact on survival in our study population. Survival did not different between patients above and below the median (p = 0.54, log rank).

## Discussion

Understanding the delicate interaction between innate and adaptive immune response at the interface between immune attack and immune suppression might help to improve immunotherapeutic approaches in CRC. In the present paper, we analyzed a potential relationship between tumor-infiltrating macrophages and dendritic cells with other immune cells and clinical parameters in CRC. We applied a broad spectrum of markers to embrace the manifoldness of different macrophage and dendritic cell subsets.

S100 is not an absolutely exclusive but reliable DC marker in CRC specimen [13,15]. Furthermore, we were able to distinguish S100-positive dendritic cells from CD163-positive macrophages by double staining for S100/CD163; this is important, since macrophages can also show weak expression of S100. Infiltration of the tumor and the stroma by macrophages and DC was found in all our specimens. However, the infiltration by macrophages or DC ranged from a very low rate of 1.7 cell/HPF up to 46/HPF. This observation reflects the broad interindividual differences, which have already been described elsewhere [15].

One primary endpoint of the present study was to investigate the relationship between tumor-infiltrating macrophages, DC and a TAA-directed systemic T-cell response. Interestingly, we found significantly increased infiltration by DC in patients without systemic T-cell response. Moreover, tumor DC infiltration strongly correlates with infiltration of regulatory T cells. It is assumed that local immature DC capture antigens, migrate to lymphoid tissue, process the antigen and, after maturation, present the antigen to specific immune cells like T cells [27]. Furthermore, we can assume that the DC found in our study are primarily immature DC, since prior studies in human CRC have shown that 1) only an mean of 1% (epithelium) and 7% (stroma) of S100+ DC were positive for the maturation marker CD208 [28] and 2) only very few CD83+CD86+ DC were found in CRC [29]. In agreement with this, we also showed in our analyses that only a small number (1/HPF) of dendritic cells are CD208-positive. Based on this knowledge about the function and presence of DC, we hypothesize that tumor stroma infiltrating immature DC primarily induce regulatory T cells as it is – with some limitations – understood as one of their key functions [30]. It remains speculative whether these Tregs may in turn reduce the induction of TAA-specific T-cell responses as we have previously shown as a weak trend [23]. However, this could serve as a reasonable explanation for the negative association between tumor-infiltrating DC (S100) and systemic TAA-directed T-cell response.

Our data confirm prior studies suggesting better survival for patients with increased DC infiltration [13-15]. We found a better survival for both high stromal and epithelial DC infiltration. A bias concerning an interaction between age, which negatively correlated with stromal DC infiltration, and survival in our study population was excluded by Kaplan-Meier analysis, which showed that survival did not differ in patients older and younger than the median. Additionally, a bias with regard to the higher S100 infiltration in lower stages was excluded by stage-corrected survival analyses, which confirmed that high DC (S100) infiltration correlates with better survival. This effect is based on data of stromal infiltration by DC.

CD11c is a marker which is expressed on myeloid DC, monocytes, and macrophages. Here, CD11c infiltration was almost 10 times lower than S100 infiltration. CD11c infiltration was increased in advanced disease and increased in patients with systemic T-cell response. Thus, CD11c-positive cells reacted differently than S100-positive cells.

During our analyses, we realized that dendritic cells expressing CD123, CD208, CD209, CD1a and Langerin do not seem to play a major role in the local immunology of human colorectal cancer, since the infiltration frequency of these CD123-positive plasmacytoid DC, DC positive for CD208 and CD209, and CD1a/Langerin-positive Langerhans cells is very low.

We used two different markers to identify tumor-infiltrating macrophages: CD68 as a less specific lysosomal marker and CD163 as a marker more specific for macrophages. CD68 + cells were the most frequent immune cell determined in the present study (about 16/HPF). Interestingly, no significant association was found between CD68+ macrophages and systemic T-cell response, Treg infiltration, and survival. Despite conflicting data in previous studies, earlier publications found better – but not significantly better – survival in CRC patients with higher macrophage infiltration [5,19].

CD163 is a macrophage/histiocyte associated scavenger receptor, which has not been analyzed in colorectal cancer thus far. An increase of CD163-positive cells in colonic mucosa seems to play a role in mediating autoimmune diseases, such as Crohn's disease [31]. Here, we found an increase of CD163-positive macrophages in limited disease and a trend for better survival in patients with high CD163 infiltration – also after correction for the stage of disease.

While CD68+ macrophages were neither increased nor decreased in limited or advanced stages, both S100 DC and CD163 macrophages were increased in the tumor stroma in limited disease (UICC I +II) compared to advanced disease (UICC III + IV).

The present study adds new data to the ongoing discussion on the interaction between innate and adoptive immune system. Our data strongly support the hypothesis that tumor-infiltrating DC are a key factor at the interface between innate and adaptive immune response in malignant disease. Tumor-infiltrating DC negatively interact with the systemic antigen-specific T-cell response but demonstrate a positive correlation with regulatory T cells and a positive association with survival in CRC. These data put tumor-infiltrating DC at the center of the relevant immune response in CRC.

## Abbreviations

CRC – colorectal cancer, Treg – regulatory T cell, MP – mononuclear phagocytes, DC dendritic cells, TAM – tumor-associated macrophages, TADC – tumor-associated dendritic cells.
